# DNA Analysis of Herbarium Specimens of the Grass Weed *Alopecurus myosuroides* Reveals Herbicide Resistance Pre-Dated Herbicides

**DOI:** 10.1371/journal.pone.0075117

**Published:** 2013-10-16

**Authors:** Christophe Délye, Chrystel Deulvot, Bruno Chauvel

**Affiliations:** INRA, UMR1347 Agroécologie, Dijon, France; University of Florence, Italy

## Abstract

Acetyl-CoA carboxylase (*ACCase*) alleles carrying one point mutation that confers resistance to herbicides have been identified in arable grass weed populations where resistance has evolved under the selective pressure of herbicides. In an effort to determine whether herbicide resistance evolves from newly arisen mutations or from standing genetic variation in weed populations, we used herbarium specimens of the grass weed *Alopecurus myosuroides* to seek mutant *ACCase* alleles carrying an isoleucine-to-leucine substitution at codon 1781 that endows herbicide resistance. These specimens had been collected between 1788 and 1975, i.e., prior to the commercial release of herbicides inhibiting ACCase. Among the 734 specimens investigated, 685 yielded DNA suitable for PCR. Genotyping the *ACCase* locus using the derived Cleaved Amplified Polymorphic Sequence (dCAPS) technique identified one heterozygous mutant specimen that had been collected in 1888. Occurrence of a mutant codon encoding a leucine residue at codon 1781 at the heterozygous state was confirmed in this specimen by sequencing, clearly demonstrating that resistance to herbicides can pre-date herbicides in weeds. We conclude that point mutations endowing resistance to herbicides without having associated deleterious pleiotropic effects can be present in weed populations as part of their standing genetic variation, in frequencies higher than the mutation frequency, thereby facilitating their subsequent selection by herbicide applications.

## Introduction

 Herbicides are synthetic organic molecules that are extremely effective in killing arable weeds by disrupting the function of proteins crucial for plant physiology (reviewed in 1). Herbicides revolutionised agriculture by tremendously facilitating weed control. Yet, whatever the herbicide target, the efficacy of the herbicides released in the field has proven to have a limited lifetime because herbicide resistance ultimately evolves in some of the weed species subjected to the herbicide selective pressure [1-3]. Mutations at the gene encoding herbicide target proteins and variation in weed response to the herbicide stress are at the root of herbicide resistance (reviewed in 1,4). Mutations at the herbicide target protein that endow resistance are well documented [2,3,5]. However, so far, not data existed allowing to determine whether some of these mutations were present in weed populations before herbicides had been released (standing genetic variation), or whether they arose after herbicide release (newly arisen variation). This question has practical implications, because if alleles carrying mutations endowing herbicide resistance exist in weed populations before herbicides are released, then the initial frequency of resistance in weed populations can be much higher than expected. Furthermore, herbicide resistance alleles pre-dating herbicide selection pressure are expected to have moderate or no associated negative pleiotropic effects on the weed life cycle (i.e., moderate or no fitness cost), and thus to be “better” resistance alleles than alleles selected by herbicide applications from *de novo* mutation [6]. 

 To determine whether an allele conferring herbicide resistance pre-dated herbicides, a possibility is to chose a weed species that evolved resistance to herbicides, then chose herbicide-resistance alleles in this species that endow no deleterious pleiotropic effects, and then seek these alleles in “naive” populations of this species, i.e., in populations that had never been subjected to herbicide selective pressure. Pleiotropic effects of the majority of herbicide resistance alleles identified to date remain to be investigated [1,7,8]. Assessment of the pleiotropic effects of herbicide resistance alleles over the whole life-cycle of a weed species has only been carried out to date for three mutant alleles of the nuclear gene encoding acetyl-coenzyme A carboxylase (ACCase) that endow resistance to herbicides targeting this enzyme in the diploid (2n = 14 [9]) grass weed *Alopecurus myosuroides* Huds. [10,11]. An *ACCase* allele carrying and isoleucine-to-leucine at codon 1781 (referred to hereafter as Leu1781 *ACCase*) had been identified as a “good” resistance allele, because it did not endow visible deleterious pleiotropic effects over *A. myosuroides* life cycle [10,11]. Leu1781 *ACCase* is thus a candidate of choice to the role of potential resistance allele pre-dating herbicide resistance in *A. myosuroides*. 

 In fields managed under “conventional” agriculture that implements herbicide applications, resistance to ACCase inhibitors is now very widespread across *A. myosuroides* geographical range [12,13]. Random population surveys identified resistance in all fields surveyed [14], and in any case, hardly any conventional fields where *A. myosuroides* is present exist where ACCase inhibitors have not been sprayed. *A. myosuroides* populations from fields cultivated under organic agriculture are also not suitable as “naive” populations: either these fields have an history of herbicide applications prior to their conversion to organic agriculture, or the *A. myosuroides* populations growing there are likely to have been contaminated by pollen flow from populations growing in neighbouring conventional fields where resistance to ACCase inhibitors had evolved [15].

 Several works have underlined the interest of plant herbaria as a highly interesting potential source of material to study ancient genetic polymorphism in plant populations (e.g., [16,17]). We therefore sought Leu1781 *ACCase* in *A. myosuroides* herbarium specimen collected before the release of herbicides. 

## Materials and Methods

### *A. myosuroides* herbarium specimen sampling

**Figure 1:**
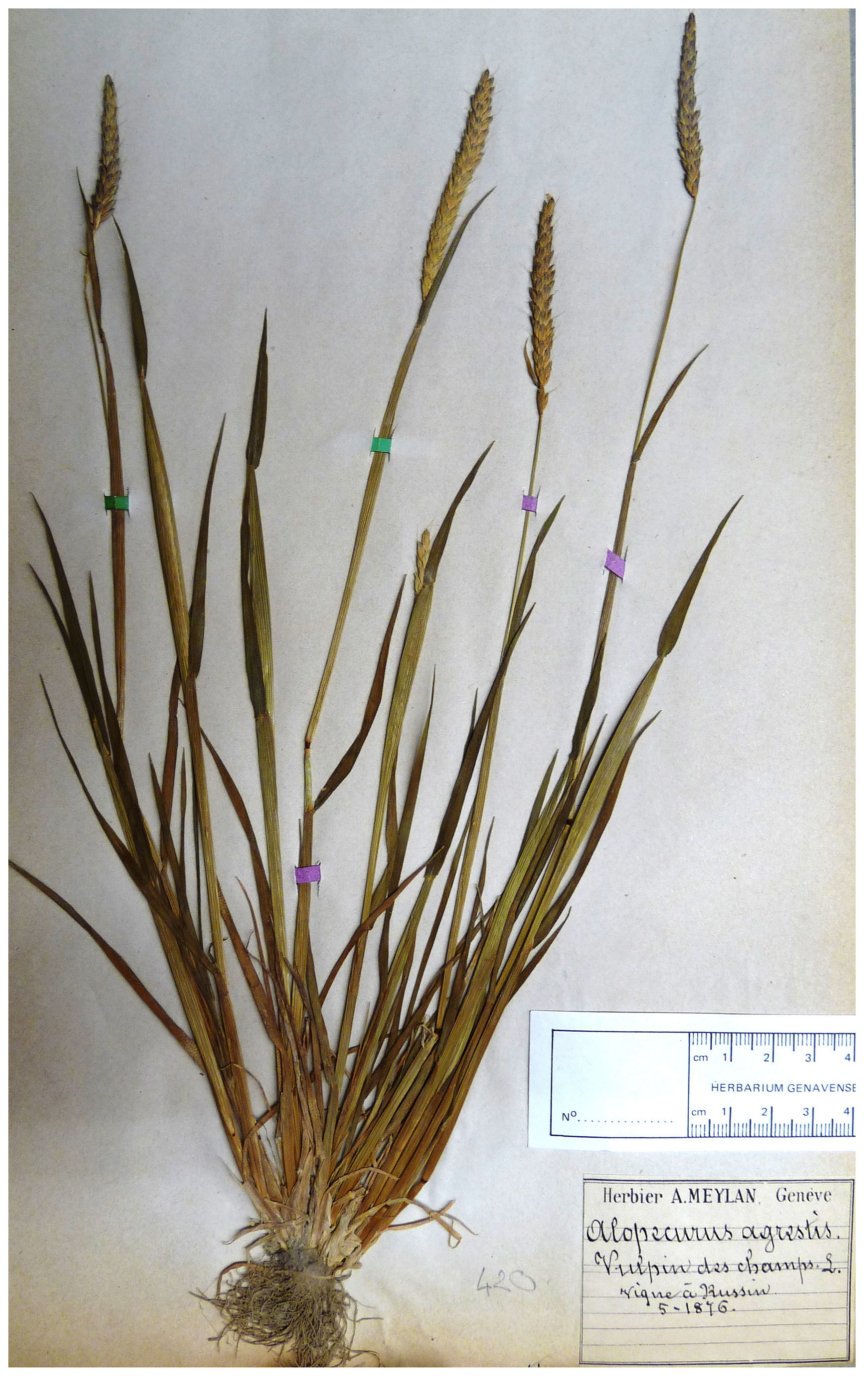
*A*. *myosuroides* herbarium specimen. Specimen collected in May 1876 in a vineyard in Russin (Switzerland), and kept at the conservatory and botanical gardens of Geneva (Switzerland). This specimen is denominated *Alopecurus* *agrestis*, a former synonym for *A*. *myosuroides*. Reprinted with permission of the copyright holder: Conservatory and Botanical Gardens of Geneva (Switzerland), 2013.

**Figure 2:**
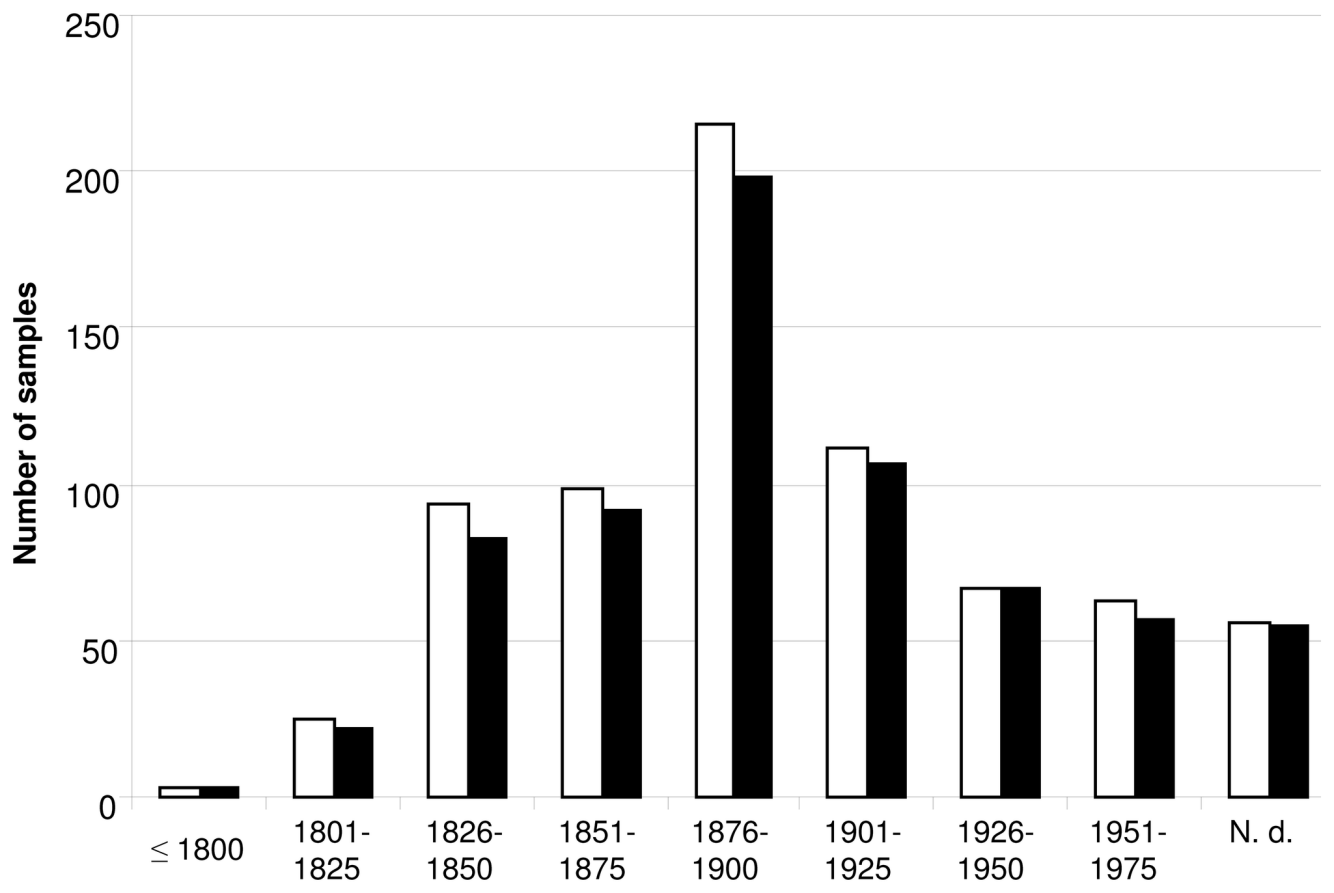
Number of samples investigated and dCAPS genotyping success per specimen collection year. White bars, number of samples collected. Black bars, number of samples successfully genotyped at ACCase codon 1781 using dCAPS. N.d., specimen collection year not determined.

 Herbarium specimens (Figure 1) were sampled in 2009 in the herbaria collections of the science museum and garden of Dijon (France), of the botanical institute of Montpellier (France), and of the conservatory and botanical gardens of Geneva (Switzerland). All necessary permissions for sampling the described specimen were obtained from the respective curators, Ms B. Remoissenet, Mr P.A. Schäfer and Mr D. Jeanmonod. A total of 734 specimens were sampled (343 in Geneva, 304 in Montpellier and 87 in Dijon), which had been collected in France (380 specimens) and in 31 other countries (Table S1). The originating country could not be determined for 96 specimens. The oldest specimen sampled had been collected in 1788, and the most recent in 1975, i.e., before ACCase-inhibiting herbicides were released in France [18]. More precisely, 428 specimens (58.3%) had been collected before 1900, and 108 (14.7%) before 1850. Collection date could not be reliably determined for 56 specimens (7.6%) (Table S1; Figure 2).

*A. myosuroides* is a grass, which makes it relatively easy to sample plant tissues on herbarium specimens without disfiguring them. One leaf (≥ 5 cm in length) was collected on each specimen using sterile gloves and disposable, sterile bistoury blades. Each leaf was placed in a paper bag labelled with a unique identifying code. Bags were closed and tape-sealed. 

### DNA extraction

 Contamination with present DNA is always a risk when working with ancient DNA [19]. To avoid contaminations by present *A. myosuroides* DNA, leaf samples collected on herbarium specimens were stored prior to DNA extraction in a microbiology laboratory in a separate building where DNA from plants had never been processed before. DNA extraction was performed in the dedicated room in this laboratory, using sterile, disposable plasticware and filtered pipette tips. Approx. 1 cm of each leaf collected on herbarium specimens was placed into a 1.5 mL microcentrifuge tube with Fontainebleau sand. The bottom of the tube was dipped in liquid nitrogen, and frozen tissues were powdered using a disposable plastic pestle. DNA was then extracted using the Wizard® Genomic DNA purification kit (Promega) following the manufacturer’s instructions, except for the last step where DNA pellets were resuspended in 50 µL rehydration solution and incubated 1h00 at 65°C prior to storage at -20°C. One blank extraction (without plant tissue) was performed in each series of 24 extractions to check for possible contaminations by present DNA.

### Genotyping using the derived Cleaved Amplified Polymorphic Sequence (dCAPS) technique

 Preliminary experiments were conducted on 30 randomly chosen DNA extracts using primers and PCR cycling programs previously described for a dCAPS assay allowing to detect Leu1781 *ACCase* [20]. The principle of dCAPS assays is to enforce a heterologous nucleotide at one or several positions in a target DNA sequence during PCR, so as to create a restriction enzyme recognition site when a specific nucleotide is present at a variable site in the sequence of the target DNA. Detection of the presence or absence of this nucleotide is achieved by digestion of the amplicon with the corresponding restriction enzyme [21]. Nineteen of the DNA samples tested did not yield any amplicon. 

 DNA from plant herbarium specimens extracted using commercial kits has proven suitable for PCR and PCR-derived applications [22,23]. However, DNA has been shown to be degraded during herbarium specimen drying and to be highly fragmented (e.g., [22,23]). Use of optimised mixes and amplification of short amplicons have been shown to improve PCR success in amplifying DNA from plant herbarium specimens [19,23-25]. In our preliminary experiment, the size of the expected amplicon was 283 nucleotides [20], which is close to the upper size limit recommended for successful PCR amplification from DNA from plant herbarium specimens (< 200-300 nucleotides; [19,23-25]). We thus designed primer ACVII27 (5’-CACAAGATGCAGCTAGATAGTGGCG) and the dCAPS primer ACVII-NspIR (5’-gccctagaatAGGCACTGGCAATAGCAGCACTTCCATGCA) to amplify a 127-nucleotide amplicon encompassing nucleotides 5257 to 5383 in the coding sequence of A. myosuroides ACCase (Genbank/EMBL accession AJ310767). This amplicon thus encompasses codons 1753 to 1794. Primer ACVII27 was fully homologous to the ACCase sequence of A. myosuroides. dCAPS primer ACVII-NspIR contained one mismatched nucleotide at its penultimate position to create a NspI recognition site specifically in amplicons carrying a wild-type sequence at ACCase codon 1781 (ATA). Design was performed following previously described rules allowing to avoid false positive detection of mutant, Leu1781 alleles (TTA or CTA) [20]. We used the Fast Cycling® PCR kit including the Q-solution (Qiagen) for PCR. DNA was added to the reaction mixes as 2 µL of the 50µL DNA solutions. Amplification was carried out as recommended in a total volume of 20µL in 0.2 mL microtubes, with primers ACVII27 and ACVII-NspIR used at a final concentration of 0.5 µM each. The cycling program consisted of 95°C 5 min followed by 40 cycles of 96°C 5 s, 58°C 5 s and 68°C 4 s, followed by a final step of 72°C 1 min. PCR mixes were assembled in the PCR-dedicated room of the microbiology laboratory mentioned before where DNA from plants had never been processed, using disposable plasticware and filtering pipette tips. The microtubes were then transferred to our laboratory without opening, and PCR programs were run on Mastercyclers (Eppendorf).

 All blank extractions were subjected to PCR like DNA samples. In each series of PCRs, at least two negative controls (PCR mixes with no DNA) were also included. All control PCRs were subsequently processed like PCRs from DNA samples.

 Digestions were performed at 37°C 3 h using 2µL of the PCR mixes added with 5 U NspI (Thermo scientific), 1 µL 10× provided enzyme buffer and 11 µL water. dCAPS patterns were visualised by electrophoresis on 3% (wt/vol) agarose gels run in 0.5× TBE buffer. Undigested amplicons (indicating the presence of a Leu1781 *ACCase* allele) had an expected size of 127 nucleotides, while the result of effective digestion (indicating the presence of an *ACCase* allele with a wild-type codon 1781 encoding an isoleucine residue) yielded two amplicons of 85 and 42 nucleotides, respectively.

 When an amplicon yielded a restriction pattern indicating the presence of a Leu1781 *ACCase* allele, the dCAPS analysis was repeated. If the same restriction pattern was obtained, the whole analysis procedure was repeated starting from the herbarium leaf fragment. If the same restriction pattern was obtained again, then an amplicon with an expected size of 175 nucleotides encompassing *A. myosuroides ACCase* codons 1753 to 1810 was generated using primers ACVII27 and ACVII34R (5’-TTCCAACAGTTCGTCCAGTAACGAATG) in six independent PCR mixes. PCR mixes and the cycling program were as before. The six PCR mixes were pooled after PCR completion, and the amplicon was sequenced on both strands using the PCR primers. 

### Statistical analyses

 Binomial confidence intervals were estimated with R [26] using Bayesian inference as implemented in the package binom [27].

## Results and Discussion

### PCR amplification success

**Figure 3:**
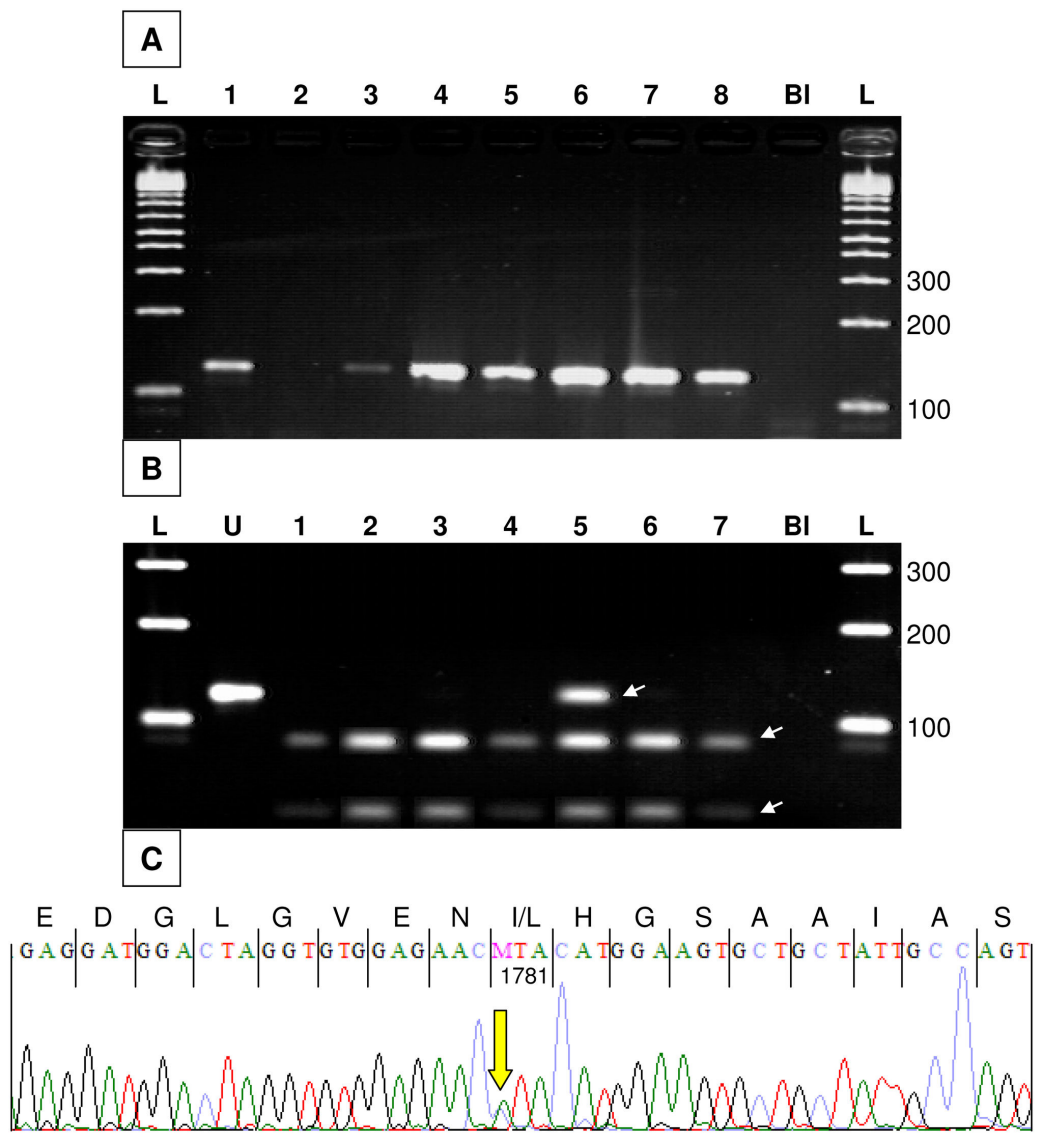
Examples of PCR amplification, dCAPS genotyping and sequencing of ACCase codon 1781 in *A*. *myosuroides* herbarium specimens. A, amplification of a 127-nucleotide amplicon from DNA extracted from *A*. *myosuroides* specimens collected in 1788 (1), 1815 (2), 1833 (3, 4), 1846 (5), 1871 (6), 1888 (7) and 1911 (8). L, DNA ladder (fragment sizes are indicated on the right); Bl, blank extraction control (PCR from a DNA extraction performed without plant material). B, dCAPS patterns obtained from *A*. *myosuroides* specimens collected in 1805 (1), 1824 (2), 1846 (3), 1871 (4), 1888 (5), 1898 (6) and 1909 (7). U, undigested amplicon (serves as a size standard); Bl, blank extraction control (dCAPS from a DNA extraction performed without plant material). The undigested, 127-nucleotide amplicon (topmost), and the digested 85-nucleotide (middle) and 42-nucleotide (bottom) amplicons are arrowed. C, Sanger sequencing chromatogram showing the sequence of codon 1781 in sample (5) from panel B. The heterozygous A (wild-type) / C (Leu1781 mutant) peak is arrowed.

 DNA was extracted from all 734 *A. myosuroides* herbarium specimens investigated. In all subsequent PCR experiments, no amplicon was obtained from blank extractions or from negative controls. No amplicon could be obtained from 49 DNA samples, even after several attempts where different combinations of starting DNA amount and primer concentration were tested. The expected amplicon was readily obtained from the remaining 685 DNA samples (exemplified on Figure 3A), i.e., an amplification success rate of 93.3% (Figure 2). Although the amplification success rate appeared lower for DNA samples extracted from specimens collected before 1851 (88.5%), success rate was not significantly dependent on the age of the herbarium specimen (Figure 2) (χ^2^=14.07; df = 8; p-value = 0.08). This is in agreement with previous studies reporting that most damage to the DNA of plant herbarium specimens that prevents PCR amplification occurs during specimen drying for preservation, and that little subsequent damage occurs during long-term storage of the specimens [22,23]. 

### Detection of the herbicide-resistant Leu1781 *ACCase* allele in one *A. myosuroides* herbarium specimen collected in 1888

 Amplicons obtained from the 685 DNA samples were submitted to NspI digestion. A mixture of digested and undigested amplicons potentially indicating the presence of a Leu1781 *ACCase* allele at the heterozygous state was observed after electrophoresis in four samples (exemplified in Figure 3B). After repeating dCAPS analysis, the mixture of digested and undigested amplicons was only clearly observed again in one DNA sample. dCAPS analysis was thus fully repeated, starting from the corresponding leaf fragment, and the mixture of digested and undigested amplicons was observed again. The PCRs performed from the corresponding blank extractions and negative controls did not yield any detectable amplicon. Sequencing was thus performed, and revealed the occurrence of an A-to-C transversion at the heterozygous state at the first position in codon 1781 (Figure 3C). Thus, out of the 685 *A. myosuroides* herbarium specimens investigated that could be analysed using dCAPS, one (0.15%) contained one Leu1781 *ACCase* allele. This specimen, kept at the Montpellier herbarium, had been collected on the 9^th^ July 1888 near Bordeaux (France) together with three other plants that did not contain Leu1781 *ACCase* (Table S1). 

 Sequence errors caused by DNA degradation have been reported when working with DNA extracted from ancient plant herbarium tissues [22]. However, the error rate observed was approx. 0.03%, errors occurred essentially in plastid DNA, and mostly consisted into C-to-T and G-to-A transitions [22]. Here, we sequenced a fragment of *ACCase* that is a nuclear gene [5], and we clearly observed an A-to-C transversion in an *A. myosuroides* herbarium specimen dating from 1888 (Figure 2C). This substitution can thus clearly not have been caused by DNA degradation. Consistent observation of independently generated dCAPS patterns all indicating the occurrence of a Leu1781 allele at the heterozygous state in this specimen further confirms our finding. As the first herbicide inhibiting ACCase used in France (diclofop) had been released in 1978 [18], i.e. 90 years after the collection of the *A. myosuroides* specimen containing a Leu1781 *ACCase* allele, our results clearly prove that herbicide resistance pre-dates herbicide use. 

### Implications

 Our work is the first demonstration ever that a mutation endowing resistance to herbicides in a weed species pre-dated herbicide discovery. Mutations endowing resistance to chemicals used to kill living organisms predating the use of such chemicals had previously only been reported in two cases. Bacteria from 30,000 year-old sediments contained genes endowing antibiotic resistance [28]. In this case, if resistance pre-dated the clinical use of antibiotics, it did not pre-date the selection pressure exerted by antibiotics, because most antibiotics currently in use are, or are derived from, compounds that have naturally been synthesised by micro-organisms for millennia. In another study more similar to our work, mutations endowing resistance to insecticides had been observed in high frequencies in pinned insect specimens collected and preserved prior to the release of these chemicals [29]. The authors could not propose a biological explanation to their findings, because the physiological function in the absence of the insecticide of protein encoded by the gene carrying the mutations was not known. 

 Exactly why Leu1781 *ACCase* pre-dated the use of herbicides inhibiting ACCase is unclear. Pre-selection of *A. myosuroides* population by natural compounds similar to herbicides inhibiting ACCase seems extremely unlikely. ACCase catalyses the first step in fatty acid and flavonoid biosynthesis [30], and is thus a vital enzyme for plant physiology. Leu1781 *ACCase* has been shown not to reduce plant vegetative and reproductive outputs in *A. myosuroides* [10,11]. This allele is fixed in several grass species [31]. In the grass *Setaria italica*, Leu1781 *ACCase* even endows increased plant vigour [32]. A putative effect on plant vigour might be an indirect reason why we observed one plant carrying Leu1781 *ACCase* allele among the 685 specimens investigated, because botanists traditionally sought healthy, vigorous plants for herbarium specimen, and the specimen carrying one Leu1781 *ACCase* allele had been collected at the margin of *A. myosuroides* geographical range in France [33], a situation where more vigorous plants are expected to better succeed. 

 Our works sheds a new light on the evolution of herbicide resistance. The observed frequency of Leu1781 *ACCase* in our sampling was one in 685 specimens. *A. myosuroides* being a diploid species [9], this corresponds to an observed frequency of Leu1781 *ACCase* of 7.3 × 10^-4^. The corresponding 95% and 99% confidence intervals are [3.65 × 10^-4^, 3.40 × 10^-3^] and [2.62 × 10^-5^, 4.67 × 10^-3^], respectively. Herbicide resistance alleles had previously been considered to have been selected from newly arisen mutations, and therefore to be initially present in weed populations at the mutation frequency, i.e., approx. 10^-9^ for an allele carrying a mutation at a given nucleotide position [34,35]. Such values seem hardly compatible with the confidence intervals computed here, which are at least four orders of magnitude higher in *A. myosuroides* specimens not selected for by herbicide applications. An alternative hypothesis would therefore be that, while some mutations endowing herbicide resistance may be present in weed populations at the nucleotide mutation frequency, possibly because of associated negative pleiotropic effects, other mutations can be present in higher frequencies as part of the weed standing genetic variation. Currently, after herbicide selection has been applied and evolution of resistance has occurred, a dozen mutant *ACCase* alleles endowing herbicide resistance have been reported in addition to Leu1781 *ACCase* [3,5]. The respective frequencies of these alleles reported in weed populations and species were substantially lower than those observed for Leu1781 *ACCase* [3,13]. The few fitness cost studies available do not allow to fully explain these differences in frequencies. Our work clearly demonstrates that Leu1781 *ACCase* alleles were part of the standing genetic variation present in *A. myosuroides* populations prior to herbicide selection. As such, Leu1781 *ACCase* alleles were most probably present in weed populations prior to herbicide selection in frequencies higher than those observed for *de novo* mutation. *ACCase* alleles endowing herbicide resistance, including Leu1781 alleles, had been shown to have evolved *via* multiple, independent mutation events [36]. Thus, applications of herbicides inhibiting ACCase on *A. myosuroides* populations would most often select Leu-1781 *ACCase*, because selection of this allele would have been faster and easier than selection of any of the other herbicide-resistant *ACCase* alleles, hence the situation observed today. 

*A. myosuroides* herbarium specimens proved invaluable to investigate and demonstrate the occurrence of alleles endowing herbicide resistance before herbicides had even been invented. Leu1781 *ACCase* was sought in herbarium specimens because, on the basis of extensive frequency assessment in *A. myosuroides* populations [13] and detailed fitness cost studies [10,11,32], we considered it the resistance allele most likely to pre-date herbicide resistance in *A. myosuroides*. With the progress of sequencing technologies, and provided enough specimens are available, herbarium specimens could also be used proactively to identify “efficient” resistance genes, i.e., genes conferring resistance without having significant deleterious pleiotropic effects, which are the genes most likely to become the most frequent and widespread in weed or pest populations, without having to conduct time-consuming fitness cost studies: “efficient” genes are likely to exist as standing genetic variation prior to pesticide selection in weed or pest populations. 

 Besides target-site resistance (i.e., mutant alleles of the gene encoding the herbicide target), herbicide resistance also includes non-target-site resistance mechanisms that are derived from weed secondary metabolism [1,4]. The current hypothesis for non-target-site resistance evolution in weeds implies selection by herbicides acting on the standing genetic variation in weed secondary metabolic pathways [1]. When alleles or mutations involved in non-target-site resistance are known, weed herbarium specimens can clearly be used to check this hypothesis.

## Supporting Information

Table S1 ***A. myosuroides* herbarium specimens analysed.** Herbarium of origin, country, locality and year of collection, and genotype at *ACCase* codon 1781 of each *A. myosuroides* specimen analysed.
(DOC)
