# Supplementary material for: DNA Analysis of Herbarium Specimens of the Grass Weed Alopecurus myosuroides Reveals Herbicide Resistance Pre-Dated Herbicides
Source: PLoS One. 2013 Oct 16;8(10):e75117. doi: 10.1371/journal.pone.0075117 (PMC3797703; doi:10.1371/journal.pone.0075117)
Supplement: Table S1 — A. myosuroides herbarium specimens analysed. Herbarium of origin, country, locality and year of collection, and genotype at ACCase codon 1781 of each A. myosuroides specimen analysed. (DOC) [file pone.0075117.s001.doc]

**Table S1 – *A. myosuroides* herbarium specimens** analysed.

| Sample | Herbarium index ^1^ | Locality ^2^ | Country ^3^ | Year ^4^ | Genotype at codon 1781 ^5^ |
| --- | --- | --- | --- | --- | --- |
| 1 | G | Dunkerque | France | 1788 | WW |
| 2 | MPU | Not specified | Not specified | 1800 | WW |
| 3 | MPU | Not specified | Not specified | 1800 | WW |
| 4 | G | Not specified | Not specified | 1805 | WW |
| 5 | G | Not specified | Not specified | 1805 | WW |
| 6 | G | Rovnye (*) | Hungary | 1807 | WW |
| 7 | G | Not specified | Not specified | 1807 | Failed |
| 8 | G | Lyon | France | 1809 | WW |
| 9 | MPU | Not specified | Not specified | 1815 | WW |
| 10 | MPU | Not specified | Not specified | 1815 | WW |
| 11 | MPU | Not specified | France | 1815 | WW |
| 12 | MPU | Maurin | France | 1816 | WW |
| 13 | MPU | Saint Biez en Belin | France | 1819 | WW |
| 14 | MPU | Not specified | Not specified | 1821 | WW |
| 15 | G | Morcoully (*) | France | 1821 | WW |
| 16 | G | Morcoully (*) | France | 1821 | Failed |
| 17 | MPU | Not specified | Not specified | 1822 | WW |
| 18 | MPU | Paris | France | 1822 | WW |
| 19 | MPU | Paris | France | 1822 | WW |
| 20 | MJSD | Not readable | Not specified | 1823 | WW |
| 21 | G | Dieuze | France | 1823 | WW |
| 22 | MPU | Not specified | Not specified | 1824 | WW |
| 23 | MPU | Not specified | Not specified | 1824 | Failed |
| 24 | MPU | Not specified | Not specified | 1824 | WW |
| 25 | MPU | Not specified | Not specified | 1824 | WW |
| 26 | MPU | Pezenas | France | 1824 | WW |
| 27 | MPU | Pezenas | France | 1824 | WW |
| 28 | G | Sceaux | France | 1825 | WW |
| 29 | MPU | Rodelle | France | 1827 | WW |
| 30 | MPU | Rodelle | France | 1827 | WW |
| 31 | G | Not specified | Not specified | 1827 | WW |
| 32 | G | Not specified | Not specified | 1827 | Failed |
| 33 | G | Aarberg | Switzerland | 1827 | WW |
| 34 | G | Not specified | Switzerland | 1827 | WW |
| 35 | MPU | Not specified | Not specified | 1828 | Failed |
| 36 | MPU | Not specified | Not specified | 1828 | WW |
| 37 | G | Genève | Switzerland | 1828 | WW |
| 38 | G | Not specified | Not specified | 1829 | WW |
| 39 | G | Epinay sous Sénart | France | 1829 | WW |
| 40 | G | Payerne | Switzerland | 1831 | WW |
| 41 | MPU | Matour | France | 1832 | WW |
| 42 | MPU | Matour | France | 1832 | WW |
| 43 | G | New Orleans | USA | 1832 | WW |
| 44 | G | Collonges | France | 1833 | WW |
| 45 | G | Vernier | Switzerland | 1833 | WW |
| 46 | G | Not specified | Not specified | 1833 | Failed |
| 47 | G | Not specified | Not specified | 1834 | WW |
| 48 | G | Arlesheim | Switzerland | 1834 | WW |
| 49 | MPU | Not readable | Not specified | 1835 | WW |
| 50 | MPU | Not specified | Not specified | 1836 | WW |
| 51 | G | Not specified | Switzerland | 1836 | WW |
| 52 | G | Not specified | Switzerland | 1836 | WW |
| 53 | MPU | Créveney | France | 1838 | WW |
| 54 | MPU | Créveney | France | 1838 | WW |
| 55 | G | Auer | Italy | 1838 | WW |
| 56 | MPU | Faye | France | 1839 | WW |
| 57 | MPU | Faye | France | 1839 | WW |
| 58 | MPU | Lattes | France | 1839 | WW |
| 59 | MPU | Lattes | France | 1839 | WW |
| 60 | MPU | Lattes | France | 1839 | WW |
| 61 | G | Not specified | Not specified | 1840 | Failed |
| 62 | G | Not specified | Not specified | 1840 | Failed |
| 63 | MPU | Lavallette | France | 1841 | WW |
| 64 | MPU | Lavallette | France | 1841 | WW |
| 65 | MPU | Lavallette | France | 1841 | WW |
| 66 | G | Not specified | Croatia | 1842 | WW |
| 67 | G | Not specified | New Zeland | 1843 | WW |
| 68 | G | Not specified | Not specified | 1843 | Failed |
| 69 | MPU | Montpellier | France | 1845 | WW |
| 70 | MPU | Not readable | Not specified | 1845 | WW |
| 71 | G | Peney | Switzerland | 1845 | WW |
| 72 | G | Genève | Switzerland | 1845 | WW |
| 73 | G | Avalia (*) | Not specified | 1845 | Failed |
| 74 | MPU | Montpellier | France | 1846 | WW |
| 75 | MPU | Montpellier | France | 1846 | WW |
| 76 | MPU | Annecy | France | 1846 | WW |
| 77 | MPU | Not specified | Germany | 1846 | WW |
| 78 | MPU | Not specified | Germany | 1846 | WW |
| 79 | MPU | Not specified | Germany | 1846 | Failed |
| 80 | MPU | Not specified | Germany | 1846 | WW |
| 81 | MPU | Not specified | Germany | 1846 | WW |
| 82 | MPU | Not specified | Germany | 1846 | Failed |
| 83 | MPU | Not specified | Germany | 1846 | WW |
| 84 | MPU | Not readable | Italy | 1846 | WW |
| 85 | MJSD | Deux Ponts | Germany | 1846 | WW |
| 86 | MJSD | Deux Ponts | Germany | 1846 | WW |
| 87 | G | Peney | Switzerland | 1846 | WW |
| 88 | G | Peney | Switzerland | 1846 | WW |
| 89 | G | Deux Ponts | Germany | 1846 | WW |
| 90 | G | Deux Ponts | Germany | 1846 | WW |
| 91 | G | Deux Ponts | Germany | 1846 | WW |
| 92 | MPU | Wimbledon | United Kingdom | 1847 | WW |
| 93 | MPU | Châteauroux | France | 1847 | WW |
| 94 | G | Genthod | Switzerland | 1847 | WW |
| 95 | MPU | Saint Raphaël | France | 1848 | WW |
| 96 | MPU | Brumath | France | 1848 | WW |
| 97 | MPU | Brumath | France | 1848 | WW |
| 98 | MPU | Brumath | France | 1848 | WW |
| 99 | MPU | Brumath | France | 1848 | WW |
| 100 | G | Brumath | France | 1848 | WW |
| 101 | G | Brumath | France | 1848 | WW |
| 102 | G | Brumath | France | 1848 | Failed |
| 103 | G | Chindrieux | France | 1848 | WW |
| 104 | G | Turin | Italy | 1848 | WW |
| 105 | G | Turin | Italy | 1848 | WW |
| 106 | G | Turin | Italy | 1848 | Failed |
| 107 | MPU | Richemont | France | 1849 | WW |
| 108 | G | Oberbaden | Germany | 1849 | WW |
| 109 | MPU | Niort | France | 1850 | WW |
| 110 | MPU | Gent | Belgium | 1850 | WW |
| 111 | MPU | Gent | Belgium | 1850 | WW |
| 112 | MPU | Gent | Belgium | 1850 | WW |
| 113 | G | Oberbaden | Germany | 1850 | WW |
| 114 | G | Vernier | Switzerland | 1850 | WW |
| 115 | G | Olave | Spain | 1850 | WW |
| 116 | G | Murcia | Spain | 1850 | WW |
| 117 | G | Marigny | France | 1850 | WW |
| 118 | G | Marigny | France | 1850 | WW |
| 119 | G | Not specified | Not specified | 1850 | WW |
| 120 | G | Paris | France | 1850 | WW |
| 121 | G | Paris | France | 1850 | WW |
| 122 | G | Paris | France | 1850 | WW |
| 123 | MPU | Not specified | France | 1851 | WW |
| 124 | G | Albertville | France | 1851 | WW |
| 125 | G | Moutiers | France | 1851 | WW |
| 126 | MPU | Bourges | France | 1852 | WW |
| 127 | MPU | Bourges | France | 1852 | WW |
| 128 | MPU | Bourges | France | 1852 | WW |
| 129 | G | Albertville | France | 1852 | WW |
| 130 | G | Albertville | France | 1852 | WW |
| 131 | G | Not specified | France | 1852 | WW |
| 132 | G | Venice | Italy | 1852 | WW |
| 133 | G | Venice | Italy | 1852 | WW |
| 134 | MPU | Not specified | France | 1853 | Failed |
| 135 | G | Albertville | France | 1853 | Failed |
| 136 | MPU | Bondy | France | 1854 | WW |
| 137 | G | Compesières | Switzerland | 1854 | WW |
| 138 | G | Genève | Switzerland | 1854 | WW |
| 139 | G | La Sarraz | Switzerland | 1855 | WW |
| 140 | G | Vernier | Switzerland | 1857 | WW |
| 141 | G | Flacey en Bresse | France | 1857 | WW |
| 142 | MPU | Autun | France | 1858 | WW |
| 143 | MPU | Autun | France | 1858 | WW |
| 144 | MJSD | Dijon | France | 1858 | WW |
| 145 | MJSD | Dijon | France | 1858 | WW |
| 146 | MJSD | Dijon | France | 1858 | WW |
| 147 | MJSD | Dijon | France | 1858 | WW |
| 148 | MJSD | Dijon | France | 1858 | WW |
| 149 | MJSD | Marsannay la Côte | France | 1858 | WW |
| 150 | MJSD | Marsannay la Côte | France | 1858 | WW |
| 151 | MJSD | Marsannay la Côte | France | 1858 | WW |
| 152 | G | Moutiers | France | 1859 | WW |
| 153 | G | Tours | France | 1859 | WW |
| 154 | G | Not specified | Switzerland | 1859 | Failed |
| 155 | G | Not specified | Switzerland | 1859 | WW |
| 156 | G | Not specified | Switzerland | 1859 | WW |
| 157 | G | Not specified | Switzerland | 1859 | WW |
| 158 | MPU | Not specified | Egypt | 1859 | WW |
| 159 | MPU | Not specified | Egypt | 1859 | WW |
| 160 | MPU | Meudon | France | 1860 | WW |
| 161 | G | Not readable | Switzerland | 1860 | WW |
| 162 | G | Cannes | France | 1861 | WW |
| 163 | G | Vernier | Switzerland | 1862 | WW |
| 164 | G | Montmorency | France | 1862 | WW |
| 165 | G | Paris | France | 1863 | WW |
| 166 | G | Paris | France | 1863 | WW |
| 167 | G | Paris | France | 1863 | WW |
| 168 | MPU | Bousquet d'Orb | France | 1864 | WW |
| 169 | MPU | Pèzenas | France | 1864 | WW |
| 170 | MPU | Uzès (Garrigues) | France | 1864 | WW |
| 171 | MPU | Verreries de Moussans | France | 1864 | WW |
| 172 | G | Not readable | France | 1864 | WW |
| 173 | G | Annecy le Vieux | France | 1865 | WW |
| 174 | G | Annecy le Vieux | France | 1865 | WW |
| 175 | G | Annecy le Vieux | France | 1865 | WW |
| 176 | G | Areuse | Switzerland | 1865 | WW |
| 177 | G | Areuse | Switzerland | 1865 | WW |
| 178 | MPU | Clermont Ferrand | France | 1866 | WW |
| 179 | MPU | Cognac | France | 1867 | WW |
| 180 | MJSD | Clermont Ferrand | France | 1867 | WW |
| 181 | MJSD | Clermont Ferrand | France | 1867 | WW |
| 182 | MJSD | Gevrey Chambertin | France | 1867 | WW |
| 183 | MJSD | Gevrey Chambertin | France | 1867 | WW |
| 184 | MPU | Not readable | Not specified | 1868 | WW |
| 185 | MPU | Not specified | Not specified | 1869 | WW |
| 186 | MPU | Not specified | Not specified | 1869 | WW |
| 187 | MPU | Sète | France | 1869 | WW |
| 188 | MJSD | Premières | France | 1869 | WW |
| 189 | G | Tripoli | Lebanon | 1869 | WW |
| 190 | G | Cartigny | Switzerland | 1869 | WW |
| 191 | G | Not specified | Switzerland | 1869 | WW |
| 192 | MPU | Lodève | France | 1870 | WW |
| 193 | MPU | Aouste sur Sye | France | 1870 | WW |
| 194 | MPU | Aouste sur Sye | France | 1870 | WW |
| 195 | MPU | Aouste sur Sye | France | 1870 | WW |
| 196 | MPU | Aouste sur Sye | France | 1870 | WW |
| 197 | G | Veyrier | Switzerland | 1870 | WW |
| 198 | MPU | Avignonet Lauragais | France | 1871 | WW |
| 199 | MPU | Avignonet Lauragais | France | 1871 | WW |
| 200 | G | Valleyres | Switzerland | 1871 | Failed |
| 201 | G | Valleyres | Switzerland | 1871 | Failed |
| 202 | MPU | Palavas les Flots | France | 1872 | WW |
| 203 | MPU | Palavas les Flots | France | 1872 | WW |
| 204 | MPU | Lattes | France | 1872 | WW |
| 205 | MPU | Lattes | France | 1872 | WW |
| 206 | G | Bex | Switzerland | 1872 | WW |
| 207 | MPU | Clermont Ferrand | France | 1873 | WW |
| 208 | MPU | Chateauneuf d'Ille et Vilaine | France | 1873 | WW |
| 209 | MPU | Chateauneuf d'Ille et Vilaine | France | 1873 | WW |
| 210 | MJSD | Saint Rémy | France | 1873 | WW |
| 211 | MJSD | Saint Rémy | France | 1873 | WW |
| 212 | MJSD | Saint Rémy | France | 1873 | WW |
| 213 | G | Genève | Switzerland | 1873 | WW |
| 214 | MPU | Montpellier | France | 1874 | WW |
| 215 | G | Genève | Switzerland | 1874 | WW |
| 216 | G | Bithyn (*) | Not specified | 1874 | WW |
| 217 | G | Lionnez (*) | Switzerland | 1875 | Failed |
| 218 | G | Zurich | Switzerland | 1875 | WW |
| 219 | G | Zurich | Switzerland | 1875 | WW |
| 220 | G | Founex | Switzerland | 1875 | Failed |
| 221 | G | Founex | Switzerland | 1875 | WW |
| 222 | MPU | Saint Martin de Londres | France | 1876 | WW |
| 223 | MPU | Saint Martin de Londres | France | 1876 | WW |
| 224 | MPU | Saint Martin de Londres | France | 1876 | WW |
| 225 | MPU | Saint Martin de Londres | France | 1876 | WW |
| 226 | MPU | Saint Martin de Londres | France | 1876 | WW |
| 227 | MPU | Saint Martin de Londres | France | 1876 | WW |
| 228 | G | Russin | Switzerland | 1876 | WW |
| 229 | MJSD | Blaisy le Bas | France | 1877 | WW |
| 230 | G | Bazoches au Houlme | France | 1877 | WW |
| 231 | G | Bayreuth | Germany | 1877 | WW |
| 232 | G | Compesières | Switzerland | 1878 | WW |
| 233 | MJSD |  | France | 1879 | WW |
| 234 | G | Vernier | Switzerland | 1879 | Failed |
| 235 | G | Montpellier | France | 1879 | WW |
| 236 | G | Beyrouth | Lebanon | 1879 | Failed |
| 237 | G | Rome | Italy | 1879 | WW |
| 238 | G | Rome | Italy | 1879 | WW |
| 239 | G | Not specified | Switzerland | 1879 | WW |
| 240 | G | Not specified | Switzerland | 1879 | WW |
| 241 | G | Cortaillod | Switzerland | 1879 | WW |
| 242 | G | Cortaillod | Switzerland | 1879 | WW |
| 243 | G | Cortaillod | Switzerland | 1879 | WW |
| 244 | MJSD | Genlis | France | 1880 | WW |
| 245 | MJSD | Auxonne | France | 1880 | WW |
| 246 | MJSD | Is sur tille | France | 1880 | WW |
| 247 | G | Bötzingen | Germany | 1880 | WW |
| 248 | G | Beyrouth | Lebanon | 1880 | WW |
| 249 | MPU | Uclès | Spain | 1881 | WW |
| 250 | G | Not specified | Sweden | 1881 | WW |
| 251 | G | Nahr el Kalb | Lebanon | 1881 | WW |
| 252 | MPU | Montlouis | France | 1882 | WW |
| 253 | MPU | Argelès | France | 1882 | WW |
| 254 | MJSD | Pouilly les Dijon | France | 1882 | WW |
| 255 | MJSD | Pouilly les Dijon | France | 1882 | WW |
| 256 | MJSD | Pouilly les Dijon | France | 1882 | WW |
| 257 | MJSD | Pouilly les Dijon | France | 1882 | WW |
| 258 | MJSD | Pouilly | France | 1882 | WW |
| 259 | MJSD | Pouilly | France | 1882 | WW |
| 260 | MJSD | Ahuy | France | 1882 | WW |
| 261 | MJSD | Ahuy | France | 1882 | WW |
| 262 | MJSD | Souhey | France | 1882 | WW |
| 263 | MJSD | Souhey | France | 1882 | WW |
| 264 | G | Hamadan | Iran | 1882 | Failed |
| 265 | G | Rascht | Iran | 1882 | WW |
| 266 | G | Rascht | Iran | 1882 | WW |
| 267 | G | Not specified | Egypt | 1882 | WW |
| 268 | G | Rolle | Switzerland | 1882 | WW |
| 269 | MPU | Saint Antoine Cumont | France | 1883 | WW |
| 270 | MPU | Saint Antoine Cumont | France | 1883 | WW |
| 271 | MPU | Aulnay | France | 1883 | WW |
| 272 | MPU | Aulnay | France | 1883 | WW |
| 273 | MPU | Aulnay | France | 1883 | WW |
| 274 | MPU | Aulnay | France | 1883 | WW |
| 275 | MPU | Renkaer (*) | Not specified | 1883 | WW |
| 276 | MPU | Renkaer (*) | Not specified | 1883 | WW |
| 277 | MPU | Aulnay | France | 1883 | WW |
| 278 | MPU | Aulnay | France | 1883 | WW |
| 279 | MPU | Aulnay | France | 1883 | WW |
| 280 | MPU | Aulnay | France | 1883 | WW |
| 281 | MPU | Saint Antoine Cumont | France | 1883 | WW |
| 282 | MPU | Saint Antoine Cumont | France | 1883 | WW |
| 283 | MPU | Saint Antoine Cumont | France | 1883 | WW |
| 284 | G | Cumont | France | 1883 | WW |
| 285 | G | Cumont | France | 1883 | WW |
| 286 | G | Aulnay | France | 1883 | WW |
| 287 | G | Aulnay | France | 1883 | WW |
| 288 | G | Aulnay | France | 1883 | WW |
| 289 | G | Aulnay | France | 1883 | WW |
| 290 | G | Aulnay | France | 1883 | Failed |
| 291 | G | Aulnay | France | 1883 | WW |
| 292 | G | Cumont | France | 1883 | WW |
| 293 | G | Renkoir | Germany | 1883 | WW |
| 294 | G | Wissembourg | France | 1883 | WW |
| 295 | G | Renkoir | Germany | 1883 | Failed |
| 296 | G | Renkoir | Germany | 1883 | WW |
| 297 | G | Not specified | Ukraine | 1885 | WW |
| 298 | G | Nassau | Bahamas | 1885 | WW |
| 299 | G | Not specified | Not specified | 1885 | WW |
| 300 | G | Dejvice | Czech Republic | 1885 | WW |
| 301 | G | Saint Petersbourg | Russia | 1885 | WW |
| 302 | G | Saint Petersbourg | Russia | 1885 | WW |
| 303 | G | Not specified | Switzerland | 1885 | Failed |
| 304 | G | Not specified | Switzerland | 1885 | Failed |
| 305 | MPU | Aniane | France | 1886 | WW |
| 306 | MPU | Grenoble | France | 1886 | WW |
| 307 | MJSD | Genlis | France | 1886 | WW |
| 308 | MJSD | Genlis | France | 1886 | WW |
| 309 | MJSD | Genlis | France | 1886 | WW |
| 310 | G | Genève | Switzerland | 1886 | WW |
| 311 | G | Montpellier | France | 1886 | WW |
| 312 | G | Not readable | Not specified | 1886 | WW |
| 313 | MPU | Béziers | France | 1887 | WW |
| 314 | MPU | Béziers | France | 1887 | WW |
| 315 | MPU | Béziers | France | 1887 | WW |
| 316 | MPU | Saint Martin de Londres | France | 1887 | WW |
| 317 | G | Oerlikon | Switzerland | 1887 | WW |
| 318 | G | Oerlikon | Switzerland | 1887 | Failed |
| 319 | MPU | Villeneuve | France | 1888 | WW |
| 320 | MPU | Villeneuve | France | 1888 | WW |
| 321 | MPU | Villeneuve | France | 1888 | WW |
| 322 | MPU | Villeneuve | France | 1888 | WW |
| 323 | MPU | Villeneuve | France | 1888 | WW |
| 324 | MPU | Villeneuve | France | 1888 | WW |
| 325 | MPU | Villeneuve | France | 1888 | WW |
| **326** | **MPU** | **Bordeaux** | **France** | **1888** | **MW** |
| 327 | MPU | Bordeaux | France | 1888 | WW |
| 328 | MPU | Caudrot | France | 1888 | WW |
| 329 | MPU | Caudrot | France | 1888 | WW |
| 330 | MPU | Caudrot | France | 1888 | WW |
| 331 | MPU | Caudrot | France | 1888 | WW |
| 332 | MPU | Not readable | Not specified | 1888 | WW |
| 333 | MPU | Not readable | Not specified | 1888 | WW |
| 334 | MPU | Bordeaux | France | 1888 | WW |
| 335 | MPU | Bordeaux | France | 1888 | WW |
| 336 | MJSD | Tailly | France | 1888 | WW |
| 337 | MJSD | Tailly | France | 1888 | WW |
| 338 | MJSD | Tailly | France | 1888 | WW |
| 339 | MJSD | Trouhans | France | 1888 | WW |
| 340 | G | Birecik | Turkey | 1888 | WW |
| 341 | MPU | Gap | France | 1889 | WW |
| 342 | MPU | Beryti | Syria | 1889 | WW |
| 343 | MPU | Bordeaux | France | 1889 | WW |
| 344 | MPU | Bordeaux | France | 1889 | WW |
| 345 | MPU | Beyrouth | Lebanon | 1889 | WW |
| 346 | G | Not specified | Turkey | 1889 | WW |
| 347 | G | Not specified | Turkey | 1889 | WW |
| 348 | G | Not specified | Turkey | 1889 | WW |
| 349 | MPU | Beziers | France | 1890 | WW |
| 350 | MPU | Beziers | France | 1890 | WW |
| 351 | MPU | Beziers | France | 1890 | WW |
| 352 | MPU | Béziers | France | 1890 | WW |
| 353 | MPU | Lattes | France | 1890 | WW |
| 354 | G | Genève | Switzerland | 1890 | WW |
| 355 | G | Riopar | Spain | 1890 | WW |
| 356 | G | Dermendere | Bulgaria | 1890 | WW |
| 357 | G | Dermendere | Bulgaria | 1890 | WW |
| 358 | MPU | Bel air | France | 1891 | WW |
| 359 | MPU | Bel air | France | 1891 | WW |
| 360 | MPU | Bel air | France | 1891 | WW |
| 361 | MPU | Castelnau | France | 1891 | WW |
| 362 | MPU | Castelnau | France | 1891 | WW |
| 363 | MPU | Castelnau | France | 1891 | WW |
| 364 | MPU | Albertville | France | 1892 | WW |
| 365 | MPU | Albertville | France | 1892 | WW |
| 366 | MPU | Albertville | France | 1892 | WW |
| 367 | MPU | Albertville | France | 1892 | WW |
| 368 | MPU | Albertville | France | 1892 | WW |
| 369 | MPU | Albertville | France | 1892 | WW |
| 370 | MPU | Albertville | France | 1892 | WW |
| 371 | G | Baltalimani | Turkey | 1892 | Failed |
| 372 | G | Villeneuve de Berg | France | 1892 | WW |
| 373 | G | Neuilly Plaisance | France | 1892 | WW |
| 374 | G | Neuilly Plaisance | France | 1892 | WW |
| 375 | G | Camarena | Spain | 1892 | WW |
| 376 | G | Camarena | Spain | 1892 | WW |
| 377 | G | Not specified | Spain | 1893 | WW |
| 378 | G | Terruel | Spain | 1893 | WW |
| 379 | G | Genève | Switzerland | 1893 | WW |
| 380 | G | Vallacloche | Spain | 1893 | WW |
| 381 | G | Vallacloche | Spain | 1893 | Failed |
| 382 | G | Vallacloche | Spain | 1893 | Failed |
| 383 | G | Bex | Switzerland | 1893 | WW |
| 384 | MJSD | Echemines | France | 1894 | WW |
| 385 | G | Cöpenirk (*) | Germany | 1894 | WW |
| 386 | G | Nassau | Bahamas | 1894 | WW |
| 387 | G | Nassau | Bahamas | 1894 | WW |
| 388 | MPU | Bordeaux | France | 1895 | WW |
| 389 | MPU | Bordeaux | France | 1895 | WW |
| 390 | MPU | Bordeaux | France | 1895 | WW |
| 391 | G | Not readable | Turkey | 1895 | WW |
| 392 | G | Perly Certoux | Switzerland | 1895 | WW |
| 393 | G | Perly Certoux | Switzerland | 1895 | WW |
| 394 | G | Not specified | Not specified | 1895 | Failed |
| 395 | MPU | Gap | France | 1896 | WW |
| 396 | MPU | Saint Germain | France | 1896 | WW |
| 397 | MJSD | Ouges | France | 1896 | WW |
| 398 | G | Not specified | Not specified | 1896 | WW |
| 399 | G | Not specified | Not specified | 1896 | WW |
| 400 | G | Paris | France | 1896 | WW |
| 401 | G | Dreux | France | 1896 | WW |
| 402 | MPU | Mauguio | France | 1897 | WW |
| 403 | MPU | Mauguio | France | 1897 | WW |
| 404 | MPU | Mauguio | France | 1897 | WW |
| 405 | MPU | Mauguio | France | 1897 | WW |
| 406 | MPU | Mauguio | France | 1897 | WW |
| 407 | MPU | Mauguio | France | 1897 | WW |
| 408 | MPU | Mauguio | France | 1897 | WW |
| 409 | MPU | Mauguio | France | 1897 | WW |
| 410 | MPU | Mauguio | France | 1897 | WW |
| 411 | G | Mauguio | France | 1897 | Failed |
| 412 | G | Not readable | Turkey | 1897 | Failed |
| 413 | G | Jaffa | Israel | 1897 | WW |
| 414 | G | Jaffa | Israel | 1897 | WW |
| 415 | MPU | Roquebrune | France | 1898 | WW |
| 416 | MPU | Roquebrune | France | 1898 | WW |
| 417 | MPU | Mauguio | France | 1898 | WW |
| 418 | MPU | Mauguio | France | 1898 | WW |
| 419 | MPU | Mauguio | France | 1898 | WW |
| 420 | MPU | Mauguio | France | 1898 | WW |
| 421 | MPU | Mauguio | France | 1898 | WW |
| 422 | MPU | Mauguio | France | 1898 | WW |
| 423 | MPU | Mauguio | France | 1898 | WW |
| 424 | G | Eyout (*) | Turkey | 1898 | WW |
| 425 | MPU | Not specified | France | 1899 | WW |
| 426 | G | Blankenloch | Germany | 1899 | WW |
| 427 | G | Koum Bachi (*) | Turkey | 1899 | Failed |
| 428 | G | Chindrieux | France | 1899 | WW |
| 429 | MPU | Montbéliard | France | 1900 | WW |
| 430 | MPU | Montbéliard | France | 1900 | WW |
| 431 | MPU | Montbéliard | France | 1900 | WW |
| 432 | MPU | Montbéliard | France | 1900 | WW |
| 433 | MPU | Storkow | Germany | 1900 | WW |
| 434 | MPU | Mauguio | France | 1900 | WW |
| 435 | G | Benguth (*) | Turkey | 1900 | Failed |
| 436 | G | Nördlingen | Germany | 1900 | WW |
| 437 | G | Serrières en Chautagne | France | 1901 | WW |
| 438 | G | Aerschot | The Netherlands | 1901 | WW |
| 439 | MJSD | Montfermeil | France | 1902 | WW |
| 440 | MJSD | Montfermeil | France | 1902 | WW |
| 441 | G | Mont (*) | France | 1902 | WW |
| 442 | G | Not readable | France | 1902 | WW |
| 443 | G | Zekeriyaköy | Turkey | 1902 | Failed |
| 444 | G | Not specified | France | 1902 | WW |
| 445 | G | Chichli (*) | Turkey | 1903 | WW |
| 446 | G | Chichli (*) | Turkey | 1903 | WW |
| 447 | G | Annecy | France | 1904 | WW |
| 448 | G | Annecy | France | 1904 | WW |
| 449 | G | Vouvray | France | 1904 | WW |
| 450 | G | Bishopsworth | United Kingdom | 1904 | WW |
| 451 | MPU | Not specified | France | 1905 | WW |
| 452 | MPU | Not specified | France | 1905 | WW |
| 453 | MJSD | Not specified | Germany | 1905 | WW |
| 454 | MJSD | Not specified | Germany | 1905 | WW |
| 455 | MJSD | Not specified | Germany | 1905 | WW |
| 456 | G | Bodman | Germany | 1905 | WW |
| 457 | G | Bodman | Germany | 1905 | Failed |
| 458 | G | Genève | Switzerland | 1905 | WW |
| 459 | G | Genève | Switzerland | 1905 | WW |
| 460 | G | Pontresina | Switzerland | 1905 | WW |
| 461 | G | Pontresina | Switzerland | 1905 | WW |
| 462 | MPU | Nazlat Zayd | Israel | 1906 | WW |
| 463 | MPU | Nazlat Zayd | Israel | 1906 | WW |
| 464 | MPU | Not specified | Not specified | 1906 | WW |
| 465 | MJSD | Königsbach | Germany | 1906 | WW |
| 466 | MJSD | Königsbach | Germany | 1906 | WW |
| 467 | MJSD | Königsbach | Germany | 1906 | WW |
| 468 | G | Allonzier la Caille | France | 1906 | WW |
| 469 | G | Allonzier la Caille | France | 1906 | WW |
| 470 | G | Königsbach | Germany | 1906 | WW |
| 471 | G | Gujuk Sou Kapalak (*) | Turkey | 1906 | WW |
| 472 | G | Königsbach | Germany | 1906 | WW |
| 473 | G | Königsbach | Germany | 1906 | WW |
| 474 | G | Riehen | Switzerland | 1906 | WW |
| 475 | G | Sutrieu | France | 1907 | WW |
| 476 | G | Sutrieu | France | 1907 | WW |
| 477 | G | Serrières en Chautagne | France | 1908 | WW |
| 478 | G | Serrières en Chautagne | France | 1908 | WW |
| 479 | G | Tours | France | 1908 | WW |
| 480 | G | Tours | France | 1908 | WW |
| 481 | G | Tours | France | 1908 | WW |
| 482 | G | Habibi (*) | Maroc | 1908 | WW |
| 483 | G | Caen | France | 1908 | WW |
| 484 | MJSD | Dieppe | France | 1909 | WW |
| 485 | MJSD | Dieppe | France | 1909 | WW |
| 486 | MJSD | Dieppe | France | 1909 | WW |
| 487 | MJSD | Myans | France | 1909 | WW |
| 488 | G | Sloupnice | Czech Republic | 1909 | WW |
| 489 | G | Gros Fourg Ruffieu | France | 1909 | WW |
| 490 | G | Gros Fourg Ruffieu | France | 1909 | WW |
| 491 | G | Gros Fourg Ruffieu | France | 1909 | WW |
| 492 | G | Gros Fourg Ruffieu | France | 1909 | WW |
| 493 | G | Not specified | Switzerland | 1909 | WW |
| 494 | G | Not readable | Not specified | 1909 | WW |
| 495 | MPU | Lattes | France | 1910 | WW |
| 496 | MPU | Lattes | France | 1910 | WW |
| 497 | MPU | Lattes | France | 1910 | WW |
| 498 | MJSD | Bordeaux | France | 1910 | WW |
| 499 | MJSD | Bordeaux | France | 1910 | WW |
| 500 | MJSD | Bordeaux | France | 1910 | WW |
| 501 | G | Jaffa | Israel | 1910 | WW |
| 502 | G | Cerdon | France | 1910 | WW |
| 503 | G | Cerdon | France | 1910 | WW |
| 504 | G | Cerdon | France | 1910 | WW |
| 505 | G | Fréjus | France | 1910 | WW |
| 506 | MPU | Lyon | France | 1910 | WW |
| 507 | MPU | Not specified | Not specified | 1910 | WW |
| 508 | MPU | Not specified | Not specified | 1910 | WW |
| 509 | MPU | Not specified | Not specified | 1910 | WW |
| 510 | G | Not specified | Israel | 1911 | WW |
| 511 | G | Ambérieu | France | 1911 | WW |
| 512 | G | Ambérieu | France | 1911 | WW |
| 513 | G | Ambérieu | France | 1911 | WW |
| 514 | G | Culoz | France | 1911 | WW |
| 515 | G | Culoz | France | 1911 | Failed |
| 516 | G | Serrières en Chautagne | France | 1911 | WW |
| 517 | G | Serrières en Chautagne | France | 1911 | WW |
| 518 | G | Serrières en Chautagne | France | 1911 | WW |
| 519 | G | Not specified | Not specified | 1911 | WW |
| 520 | G | Cartigny | Switzerland | 1912 | WW |
| 521 | G | Cartigny | Switzerland | 1912 | WW |
| 522 | MPU | La Prairie | France | 1913 | WW |
| 523 | MPU | La Prairie | France | 1913 | WW |
| 524 | G | Ruffieu | France | 1913 | WW |
| 525 | G | Genève | Switzerland | 1913 | Failed |
| 526 | G | Genève | Switzerland | 1913 | WW |
| 527 | G | Genève | Switzerland | 1913 | WW |
| 528 | G | Genève | Switzerland | 1913 | WW |
| 529 | G | Annandale | USA | 1914 | WW |
| 530 | G | Chambéry | France | 1916 | WW |
| 531 | G | Chambéry | France | 1916 | WW |
| 532 | MPU | Vaux | Not specified | 1917 | WW |
| 533 | MPU | Salonique | Greece | 1918 | WW |
| 534 | MPU | Salonique | Greece | 1918 | WW |
| 535 | MPU | Cordes sur Ciel | France | 1918 | WW |
| 536 | MPU | Not readable | Not specified | 1919 | WW |
| 537 | MPU | Not readable | Not specified | 1919 | WW |
| 538 | MPU | Not readable | Not specified | 1919 | WW |
| 539 | MPU | Bordeaux | France | 1921 | WW |
| 540 | MPU | Bordeaux | France | 1921 | WW |
| 541 | MPU | Saint Martin de Londres | France | 1921 | WW |
| 542 | MPU | Saint Martin de Londres | France | 1921 | Failed |
| 543 | G | Not specified | Not specified | 1921 | WW |
| 544 | MPU | Valras la plage | France | 1922 | WW |
| 545 | MPU | Valras la plage | France | 1922 | WW |
| 546 | MPU | Valras la plage | France | 1922 | WW |
| 547 | MPU | Montpellier | France | 1922 | WW |
| 548 | MPU | Salindres | France | 1924 | WW |
| 549 | MPU | Not specified | Germany | 1926 | WW |
| 550 | MPU | Not specified | France | 1926 | WW |
| 551 | G | Not specified | Sweden | 1926 | WW |
| 552 | G | Not specified | Sweden | 1926 | WW |
| 553 | G | Collonges | France | 1926 | WW |
| 554 | G | Collonges | France | 1926 | WW |
| 555 | G | Binningen | Switzerland | 1926 | WW |
| 556 | MPU | Luxembourg | France | 1928 | WW |
| 557 | MPU | Iven | Germany | 1928 | WW |
| 558 | MPU | Iven | Germany | 1928 | WW |
| 559 | MPU | Iven | Germany | 1928 | WW |
| 560 | MPU | Iven | Germany | 1928 | WW |
| 561 | MPU | Iven | Germany | 1928 | WW |
| 562 | MPU | Salindres | France | 1928 | WW |
| 563 | MJSD | Saulx le Duc | France | 1928 | WW |
| 564 | MJSD | Saulx le Duc | France | 1928 | WW |
| 565 | G | Les Marches | France | 1928 | WW |
| 566 | MPU | Palavas les flots | France | 1929 | WW |
| 567 | G | Bilecik | Turkey | 1929 | WW |
| 568 | G | Bilecik | Turkey | 1929 | WW |
| 569 | MPU | Nancy | France | 1930 | WW |
| 570 | MJSD | Flavigny sur Ozerain | France | 1930 | WW |
| 571 | G | Not specified | Switzerland | 1931 | WW |
| 572 | G | Not specified | Switzerland | 1931 | WW |
| 573 | G | Genève | Switzerland | 1932 | WW |
| 574 | MPU | Baillargues | France | 1933 | WW |
| 575 | MPU | Baillargues | France | 1933 | WW |
| 576 | MPU | Baillargues | France | 1933 | WW |
| 577 | MPU | Baillargues | France | 1933 | WW |
| 578 | MPU | Galargues | France | 1934 | WW |
| 579 | MPU | Galargues | France | 1934 | WW |
| 580 | MPU | Savonnières | France | 1934 | WW |
| 581 | MPU | Savonnières | France | 1934 | WW |
| 582 | MPU | Not specified | Germany | 1934 | WW |
| 583 | MPU | Not specified | Germany | 1934 | WW |
| 584 | MPU | Not specified | Germany | 1934 | WW |
| 585 | MPU | Not specified | Germany | 1934 | WW |
| 586 | MPU | Luxembourg | Luxembourg | 1935 | WW |
| 587 | MJSD | Mesmont | France | 1935 | WW |
| 588 | MJSD | Mesmont | France | 1935 | WW |
| 589 | MPU | Poigny | France | 1936 | WW |
| 590 | MPU | Poigny | France | 1936 | WW |
| 591 | MJSD | Vielverge | France | 1936 | WW |
| 592 | G | Poigny | France | 1936 | WW |
| 593 | MPU | Savonnières | France | 1937 | WW |
| 594 | MPU | Savonnières | France | 1937 | WW |
| 595 | MJSD | Yzeure | France | 1937 | WW |
| 596 | MJSD | Yzeure | France | 1937 | WW |
| 597 | G | Not specified | United Kingdom | 1937 | WW |
| 598 | G | Fouar | Lebanon | 1937 | WW |
| 599 | MPU | Berthenay | France | 1938 | WW |
| 600 | MPU | Berthenay | France | 1938 | WW |
| 601 | MPU | Saulx le Vesoul | France | 1938 | WW |
| 602 | G | Antioche | Turkey | 1938 | WW |
| 603 | G | Not specified | Switzerland | 1938 | WW |
| 604 | G | Kalkali (*) | Turkey | 1939 | WW |
| 605 | G | Ergani | Turkey | 1940 | WW |
| 606 | G | Not specified | Greece | 1942 | WW |
| 607 | G | Sitia | Greece | 1942 | WW |
| 608 | G | Arlesheim | Switzerland | 1943 | WW |
| 609 | G | Vandoeuvres | Switzerland | 1945 | WW |
| 610 | G | Not specified | United Kingdom | 1946 | WW |
| 611 | G | Dardagny | Switzerland | 1948 | WW |
| 612 | MPU | Not specified | France | 1948 | WW |
| 613 | MJSD | Saulon la Rue | France | 1950 | WW |
| 614 | G | Limburg | The Netherlands | 1950 | WW |
| 615 | G | Becceril | Spain | 1950 | WW |
| 616 | MJSD | Pourcy | France | 1951 | WW |
| 617 | MJSD | Pourcy | France | 1951 | WW |
| 618 | G | Kafer Shams? | Syria | 1951 | WW |
| 619 | G | Ghouta | Syria | 1951 | WW |
| 620 | G | Damas | Syria | 1951 | WW |
| 621 | MJSD | Orléans | France | 1952 | WW |
| 622 | G | Not specified | Switzerland | 1952 | WW |
| 623 | G | Sion | Switzerland | 1952 | WW |
| 624 | G | Sion | Switzerland | 1952 | WW |
| 625 | G | Ghouta | Syria | 1952 | Failed |
| 626 | G | Isir dech Choghour (*) | Not specified | 1952 | WW |
| 627 | G | Nabr el Kelb (*) | Not specified | 1952 | WW |
| 628 | G | Baghdad | Iraq | 1952 | WW |
| 629 | G | Bagdad | Iraq | 1952 | WW |
| 630 | MJSD | La veuve | France | 1953 | WW |
| 631 | G | Homs | Syria | 1953 | WW |
| 632 | MJSD | Châtillon Coligny | France | 1954 | WW |
| 633 | G | karamanöglu | Turkey | 1954 | WW |
| 634 | MJSD | Villeberny | France | 1955 | WW |
| 635 | G | Er Radd (*) | Not specified | 1955 | WW |
| 636 | G | Nahariya | Israel | 1955 | WW |
| 637 | G | Débogh Khané (*) | Not specified | 1956 | Failed |
| 638 | G | Débogh Khané (*) | Not specified | 1956 | WW |
| 639 | G | Débogh Khané (*) | Not specified | 1956 | Failed |
| 640 | G | Débogh Khané (*) | Not specified | 1956 | WW |
| 641 | G | Débogh Khané (*) | Not specified | 1956 | Failed |
| 642 | G | Débogh Khané (*) | Not specified | 1956 | WW |
| 643 | G | Débogh Khané (*) | Not specified | 1956 | Failed |
| 644 | MPU | Not specified | France | 1957 | WW |
| 645 | G | Maqil (*) | Iraq | 1957 | WW |
| 646 | G | Maqil (*) | Iraq | 1957 | WW |
| 647 | G | Karmat el Nabi (*) | Iraq | 1957 | WW |
| 648 | G | Erbil | Iraq | 1957 | WW |
| 649 | G | Tercan | Turkey | 1957 | WW |
| 650 | G | Abu Ghrahib | Iraq | 1957 | Failed |
| 651 | G | Abu Ghrahib | Iraq | 1957 | WW |
| 652 | G | Abu Ghrahib | Iraq | 1957 | WW |
| 653 | G | Taagense | Denmark | 1959 | WW |
| 654 | G | Kaliartum (*) | Greece | 1959 | WW |
| 655 | G | Grézieux le Fromental | France | 1959 | WW |
| 656 | G | Not readable | United Kingdom | 1960 | WW |
| 657 | MPU | Not readable | Not specified | 1960 | WW |
| 658 | MPU | Not specified | France | 1960 | WW |
| 659 | MPU | Not specified | France | 1960 | WW |
| 660 | G | La Plaine | Switzerland | 1961 | WW |
| 661 | G | Lully | Switzerland | 1961 | WW |
| 662 | G | Chassenard | France | 1961 | WW |
| 663 | G | Ispahan | Iran | 1962 | WW |
| 664 | G | Ispahan | Iran | 1962 | WW |
| 665 | G | Mazandaran | Iran | 1963 | WW |
| 666 | G | Chancy | Switzerland | 1965 | WW |
| 667 | G | Kefraya (*) | Lebanon | 1965 | WW |
| 668 | MPU | Allevard | France | 1967 | WW |
| 669 | MJSD | Erans | France | 1969 | WW |
| 670 | G | Fâryâb | Afghanistan | 1971 | WW |
| 671 | G | Fâryâb | Afghanistan | 1971 | WW |
| 672 | G | Kondôz | Afghanistan | 1971 | WW |
| 673 | G | Alishang | Afghanistan | 1971 | WW |
| 674 | G | Alishang | Afghanistan | 1971 | WW |
| 675 | G | Not specified | Afghanistan | 1971 | WW |
| 676 | G | Darüntha | Afghanistan | 1971 | WW |
| 677 | G | Aynak | Afghanistan | 1972 | WW |
| 678 | G | Not specified | Azerbaijan | 1975 | WW |
| 679 | MPU | Not specified | France | Not specified | WW |
| 680 | MPU | Not specified | France | Not specified | WW |
| 681 | MPU | Not specified | France | Not specified | Failed |
| 682 | MPU | Canet en Roussillon | France | Not specified | WW |
| 683 | MPU | Canet en Roussillon | France | Not specified | WW |
| 684 | MPU | Perpignan | France | Not specified | WW |
| 685 | MPU | Savoye | France | Not specified | WW |
| 686 | MPU | Montpellier | France | Not specified | WW |
| 687 | MPU | Not specified | Not specified | Not specified | WW |
| 688 | MPU | Not specified | Sweden | Not specified | WW |
| 689 | MPU | Not specified | Sweden | Not specified | WW |
| 690 | MPU | Dreux | France | Not specified | WW |
| 691 | MPU | Chateaudun | France | Not specified | WW |
| 692 | MPU | Chateaudun | France | Not specified | WW |
| 693 | MPU | Not specified | Not specified | Not specified | WW |
| 694 | MPU | Not specified | Not specified | Not specified | WW |
| 695 | MPU | Not specified | Not specified | Not specified | WW |
| 696 | MPU | Not specified | Not specified | Not specified | WW |
| 697 | MPU | Not specified | Not specified | Not specified | WW |
| 698 | MPU | Not specified | Not specified | Not specified | WW |
| 699 | MPU | Saint Rémy | France | Not specified | WW |
| 700 | MPU | Heidelberg | Germany | Not specified | WW |
| 701 | MPU | Not specified | Not specified | Not specified | WW |
| 702 | MPU | Not specified | France | Not specified | WW |
| 703 | MPU | Not specified | Not specified | Not specified | WW |
| 704 | MPU | Not specified | Not specified | Not specified | WW |
| 705 | MPU | Not specified | Not specified | Not specified | WW |
| 706 | MPU | Not specified | Not specified | Not specified | WW |
| 707 | MPU | Not specified | Not specified | Not specified | WW |
| 708 | MPU | Not specified | Not specified | Not specified | WW |
| 709 | MPU | Not specified | Not specified | Not specified | WW |
| 710 | MPU | Not specified | Not specified | Not specified | WW |
| 711 | MPU | Batoumi | Georgia | Not specified | WW |
| 712 | MPU | Batoumi | Georgia | Not specified | WW |
| 713 | MPU | Batoumi | Georgia | Not specified | WW |
| 714 | MPU | Not specified | Not specified | Not specified | WW |
| 715 | MPU | Not specified | Not specified | Not specified | WW |
| 716 | MPU | Paris | France | Not specified | WW |
| 717 | MPU | Marin | France | Not specified | WW |
| 718 | MPU | Saint Sever | France | Not specified | WW |
| 719 | MPU | Not specified | Not specified | Not specified | WW |
| 720 | MJSD | Not specified | Not specified | Not specified | WW |
| 721 | MJSD | Velars sur Ouche | France | Not specified | WW |
| 722 | MJSD | Not specified | France | Not specified | WW |
| 723 | MJSD | Not specified | France | Not specified | WW |
| 724 | MJSD | Not specified | France | Not specified | WW |
| 725 | MJSD | Not specified | France | Not specified | WW |
| 726 | MJSD | Not readable | Not specified | Not specified | WW |
| 727 | MJSD | Not specified | Not specified | Not specified | WW |
| 728 | MJSD | Not specified | Not specified | Not specified | WW |
| 729 | MJSD | Not specified | Not specified | Not specified | WW |
| 730 | MJSD | Not specified | Not specified | Not specified | WW |
| 731 | MJSD | Not specified | France | Not specified | WW |
| 732 | MJSD | Not specified | France | Not specified | WW |
| 733 | G | Geneva | Switzerland | Not specified | WW |
| 734 | G | Not specified | Not specified | Not specified | WW |

^1^ G, Geneva (Switzerland); MJSD, Dijon (France); MPU, Montpellier (France) according to the Index Herbariorum (http://sciweb.nybg.org/science2/IndexHerbariorum.asp).

^2^ (*), label reading unsure.

^3^ Country names as indicated on the label. Because of historical events, some locations may currently be located in other or in new countries.

^4^ Year the herbarium specimen had been collected, as specified in the annotations to the specimen.

^5^ Genotype at codon 1781: WW, homozygous wild-type; MW, heterozygous mutant (i.e., contains one Leu1781 ACCase allele); Failed, no amplicon could be obtained from DNA extracted from the herbarium specimen tissue.
